# Immobilizing lead and copper in aqueous solution using microbial- and enzyme-induced carbonate precipitation

**DOI:** 10.3389/fbioe.2023.1146858

**Published:** 2023-03-27

**Authors:** Lin Wang, Wen-Chieh Cheng, Zhong-Fei Xue, Md Mizanur Rahman, Yi-Xin Xie, Wenle Hu

**Affiliations:** ^1^ School of Civil Engineering, Xi’an University of Architecture and Technology, Xi’an, China; ^2^ Shaanxi Key Laboratory of Geotechnical and Underground Space Engineering (XAUAT), Xi’an, China; ^3^ UniSA STEM, SIRM, University of south Australia, Adelaide, SA, Australia

**Keywords:** MICP, EICP, heavy metal, remediation efficiency, thermodynamic properties

## Abstract

Inappropriate irrigation could trigger migration of heavy metals into surrounding environments, causing their accumulation and a serious threat to human central nervous system. Traditional site remediation technologies are criticized because they are time-consuming and featured with high risk of secondary pollution. In the past few years, the microbial-induced carbonate precipitation (MICP) is considered as an alternative to traditional technologies due to its easy maneuverability. The enzyme-induced carbonate precipitate (EICP) has attracted attention because bacterial cultivation is not required prior to catalyzing urea hydrolysis. This study compared the performance of lead (Pb) and copper (Cu) remediation using MICP and EICP respectively. The effect of the degree of urea hydrolysis, mass and species of carbonate precipitation, and chemical and thermodynamic properties of carbonates on the remediation efficiency was investigated. Results indicated that ammonium ion (NH_4_^+^) concentration reduced with the increase in lead ion (Pb^2+^) or copper ion (Cu^2+^) concentration, and for a given Pb^2+^ or Cu^2+^ concentration, it was much higher under MICP than EICP. Further, the remediation efficiency against Cu^2+^ is approximately zero, which is way below that against Pb^2+^ (approximately 100%). The Cu^2+^ toxicity denatured and even inactivated the urease, reducing the degree of urea hydrolysis and the remediation efficiency. Moreover, the reduction in the remediation efficiency against Pb^2+^ and Cu^2+^ appeared to be due to the precipitations of cotunnite and atacamite respectively. Their chemical and thermodynamic properties were not as good as calcite, cerussite, phosgenite, and malachite. The findings shed light on the underlying mechanism affecting the remediation efficiency against Pb^2+^ and Cu^2+^.

## 1 Introduction

Metallurgical processes, smelting activities, and inappropriate irrigation can discharge heavy metals into surrounding environments, and their accumulation can badly cause damage to the liver and kidney function of human body (Chen et al., 2022; Bai et al., 2021a; Chen et al., 2023; Bai et al., 2021b; Wen et al., 2023). Lead (Pb) and copper (Cu) are considered two often-seen contaminants because of their non-biodegradability and bioaccumulation (Bai et al., 2022b; Bai et al., 2022c; Xue et al., 2023; Xue et al., 2021; Xie et al., 2023; Wang et al., 2022c). Transforming heavy metals from the solid phase to the solution phase by their mobility or solubility increases notably their bioavailability. To this end, immobilising Pb and Cu is deemed crucial in securing the safety of surrounding environments and human health (Khadim et al., 2019; Wang et al., 2021; Xue et al., 2022). Soil flushing (Tampouris et al., 2001; Dermont et al., 2008; Zhu et al., 2021), electrokinetic remediation (Lockhart, 1983; Mena et al., 2015; Liu et al., 2018; Wang et al., 2023b), chemical precipitation (Khan et al., 2004; Xia et al., 2019; Gong et al., 2020), ion exchange (Hamby, 1996; Aparicio et al., 2021; Odom et al., 2021), and phytoremediation (Jalali and Khanlari, 2007; Sylvain et al., 2016; Zine et al., 2020) have been widely applied to tackle the raised issue. Notwithstanding that, their development and application are impeded because they are usually time-consuming and can impose risks of secondary pollution (Bhattacharya et al., 2018; Hu et al., 2021a; Hu et al., 2021b).

Bioprecipitation of calcium carbonate has gained increasing attention (Jarwar et al., 2022; Omoregie et al., 2022; Yang et al., 2020). As the name suggests, bioprecipitation introduces microorganisms, especially bacteria. The pathway of bioprecipitation mainly includes numerous mechanisms, including urea hydrolysis, denitrification, iron reduction, and sulfate reduction. Urea hydrolysis is one of the most efficient, economic pathways to implement bioprecipitation. During bioprecipitation, the urea hydrolysis-induced metabolites (e.g., carbonate ions) react with metals present in the wastewater or soils and form metal precipitates. That is to say, it converts the metals from its aqueous phase into a solid phase, reducing the potential of migration for metals (Zhu and Maria, 2016; Zhang et al., 2020; Yuan et al., 2021; Hu et al., 2022).

Biologically controlled mineralization and biologically induced mineralization are the most common methods of biomineralization. The microbial-induced carbonate precipitation (MICP) (Castanier et al., 1993; Gollapudi et al., 1995; Phillips et al., 2016; Torres-Aravena et al., 2018) and the enzyme-induced carbonate precipitation (EICP) (Maubois, 1984; Larsen et al., 2008; Ashkan et al., 2019; Moghal et al., 2020) belong to the latter. *Sporosarcina pasteurii* due to its extremely high activity has extensively been used as ureolytic bacteria for catalysing urea hydrolysis. Jiang et al. (2019) declared that bacterial concentration and calcium source can impact the remediation efficiency which is defined as the ratio of the removed heavy metal concentration to the initial concentration. There are three inherent mechanisms that play a major part in the biomineralization process, including abiotic precipitation, biosorption, and biotic precipitation. It is well acknowledged that the abiotic precipitation could badly degrade the remediation efficiency because of its low thermodynamic stability. Further, the higher the degree of urea hydrolysis, the more the carbonate ions precipitated with heavy metals, and the higher the remediation efficiency (Achal et al., 2012; Xue et al., 2022). However, Duarte-Nass et al. (2020) found that a low remediation efficiency against Cu also appears when subjected to higher degrees of urea hydrolysis. Moreover, considering calcium ion (Ca^2+^) forms competitive adsorption with heavy metal ions, the ureolytic bacteria bind preferentially themselves with Ca^2+^, indicating an enhancement of the resistance against heavy metal ions. An inappropriate calcium source could lead to a change in surrounding pH towards affecting the remediation of heavy metals (Wen et al., 2019; Wang et al., 2022b). Under CaO, its reaction with H_2_O forms Ca(OH)_2_ and then notably elevates the surrounding pH. Such high pH badly depresses the urease activity, reducing the ammonium ion (NH_4_^+^) concentration (Wang et al., 2022a). The pH is measured as the lowest under Ca(CH_3_COO)_2_, causing the degradation of carbonate precipitation (Wang et al., 2022a). There are other microbial methods available in recent years. A phosphate-solubilizing strain of *Pseudomonas* sp. was isolated from a phosphate mining wasteland and applied to solubilize phosphate rock and immobilize Pb (Wang et al., 2020; Xiao et al., 2021; Li et al., 2022). Results showed that a number of functional groups on the phosphate rock surface and *Pseudomonas* sp. was amended, and *Pseudomonas* sp. could form hydroxyapatite and pyrophosphate with Pb ions.

Further, enzymes are more environmentally adaptive when compared to microorganisms that require appropriate environments for living and supply of oxygen and nutrients. Moreover, nanometer-sized enzymes are much smaller than micrometer-sized microorganisms, and therefore, they can penetrate into the deeper grounds with no difficulty when applied to ‘*in-situ*’ conditions. Li et al. (2022) explored the inherent mechanisms affecting the retention of cadmium ion (Cd^2+^). Cd^2+^ were immobilised with otavite (CdCO_3_), calcite co-precipitation (CaCO_3_-Cd), and vaterite/aragonite chemisorption (CaMg(CO_3_)_2_). Despite that, the above analysis reveals several gaps and shortcomings that remain to be addressed in the future. Comparison between MICP and EICP has not been conducted yet concerning multiple perspectives such as the degree of urea hydrolysis, the precipitation mass, and species of carbonate precipitation. Furthermore, carbonate precipitation of low chemical and thermodynamic properties may dissolve or degrade when subjected to harsh conditions. This part is neglected in a large body of research and is worthy of investigation. The above may apply to explore the inherent mechanism affecting the removal of Pb and Cu. The main objectives of this study are: 1) to conduct a comparison of the degree of urea hydrolysis, and mass and species of carbonate precipitation between MICP and EICP, 2) to investigate the chemical and thermodynamic properties of carbonates, and 3) to reveal the inherent mechanisms affecting the removal of Pb and Cu.

## 2 Materials and methods

### 2.1 MICP: Bacteria and cultivation

The use of *Sporosarcina pasteurii* in a freeze-dried form aimed to catalyse urea hydrolysis. The bacterial strain was brought back to room temperature in the first place. The strain of 0.1 mL was transferred to a 100 mL liquid medium composed of NH_4_Cl (Chengdu Chron Chemicals Co., Ltd., China) of 10 g/L, urea (Damao Chemical Reagent Factory, China) of 20 g/L, yeast extract (Oxoid Ltd., United Kingdom) of 10 g/L, MnSO_4_·H_2_O (Shanghai Aladdin Biochemical Technology Co., Ltd., China) of 10 mg/L, and NiCl·6H_2_O (Tianli Chemical Reagent Co., Ltd., China) of 24 mg/L for their cultivation at 30°C and at 180 rpm for 24 h toward reactivating the bacterial strain. The chemicals are analytically pure. Furthermore, pH of the bacteria resuscitation environment being 8.8 was measured using a benchtop pH meter (HI 2003; HANNA Instruments Inc., Italy). The bacterial solution was mixed with glycerol using a 7:3 ratio and stored at −20°C for future use.

The biomass (OD_600_) and urease activity (UA) were measured for a 172-h period through the activated ureolytic bacteria (i.e., *Sporosarcina pasteurii*) when subjected to pH values of 6, 8, 8.8, and 10 respectively. OD_600_ was measured by a visible light spectrophotometer (721 G; Inesa Analytical Instrument Co., LTD., China). For the sake of brevity, the biomass and the optimal ratio of the culture medium are not presented here and can refer to the authors’ published work (Xue et al., 2022). The measurement of UA was on a basis of the ureolysis rate and referred to the method recommended by Whiffin et al. (2007); 2 mL bacterial culture or plant enzyme is mixed with 18 mL 1.11 M urea and the electrical conductivity (EC) is measured at 0 and 5 min by a benchtop conductivity meter (HI2314; HANNA Instruments Inc., Italy). Eqs. 1, 2 show the equation applied to UA and specific urease activity (SUA) evaluation. UA under MICP is measured being 18.83 mM urea hydrolyzed min^-1^. OD_600_ is measured as 1.8–2.1. SUA is calculated as 8.97–10.46 mM urea hydrolyzed min^-1^ OD_600_^−1^.

$$UA=\frac{{EC}_{5}-{EC}_{0}}{5}\times 10\times 1.11\;\left(mM\;urea\;hydrolyzed\;\min^{-1}\right)$$ (1)

$$SUA=\frac{UA}{{OD}_{600}}\;\left(mM\;urea\;hydrolyzed\;\min^{-1}{{OD}_{600}}^{-1}\right)$$ (2)

where *EC*_0_ and *EC*_5_ are the electrical conductivity at 0 and 5 min respectively. A higher UA represents a higher resistance against heavy metal stress and is likely to achieve a higher remediation efficiency.

### 2.2 MICP: Test tube experiments

**FIGURE 1:**
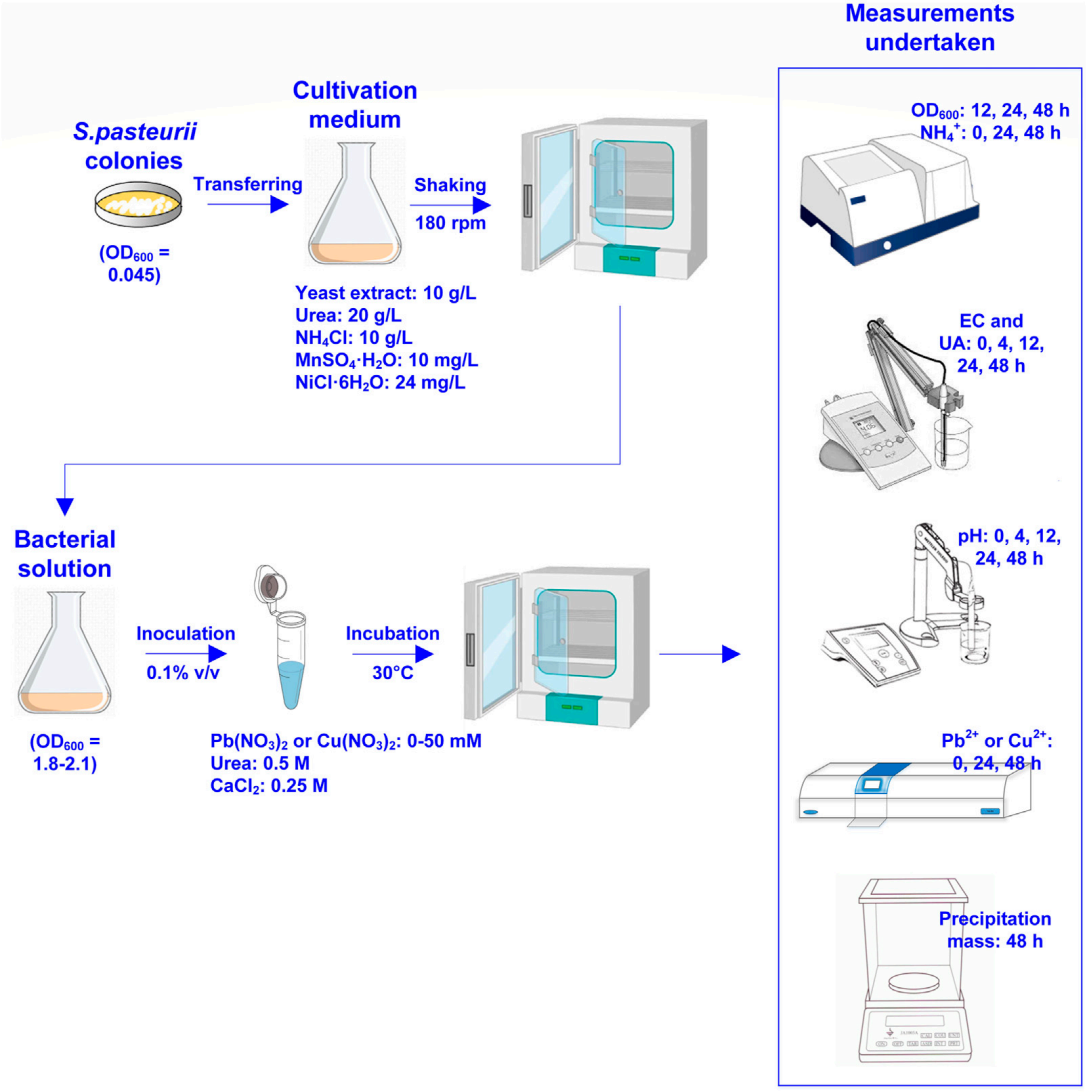
Flowchart of the test tube experiments applied to Pb or Cu remediation using the microbial-induced carbonate precipitation.

In the present work, MICP was attained through a series of test tube experiments and applied to the removal of Pb and Cu. The OD_600_ curve determined how long did the cultivation take the ureolytic bacteria to achieve their highest activity. The blank OD_600_ was measured to be 0.045 prior to the use of the bacterial solution. As recommended by Duarte-Nass et al. (2020), 0.33 M is considered as the minimum urea concentration that is required to promote *Sporosarcina pasteurii* to grow and reproduce. 0.5 M urea concentration was, therefore, adopted herein. The concentration of calcium source was set to a value five times higher than the contaminant concentration (Fang et al., 2021). A significant body of research takes the concentration of heavy metals in aqueous solution below 5 mM and those in soils below 400 mg/kg into account (He et al., 2019; Fang et al., 2021; Liu et al., 2021). A 5–50 mM range of lead ion (Pb^2+^) or copper ion (Cu^2+^) concentration is applied to the present work and aims to investigate not only the variation of the degree of urea hydrolysis with Pb^2+^ or Cu^2+^ concentration but the change in the species of carbonate precipitation. Given a maximum of 50 mM applied to Pb^2+^ or Cu^2+^ concentration, the concentration of calcium source was thus set to 0.25 M. Upon the completion of bacterial cultivation, the bacterial solution with OD_600_ values falling within a 1.8–2.1 range was inoculated (10% (v/v)) into the liquid medium containing Pb(NO_3_)_2_ or Cu(NO_3_)_2_ at concentrations varying in a 0–50 mM range, 0.5 M urea, 0.25 M CaCl_2_, and 2 g/L yeast extract. In the present work, three replicates were considered for each test set. The results were expressed as arithmetic means with standard deviations. The data means were compared using Fisher’s least significant difference (LSD) method, and the significant difference was set at 0.05. EC, pH, and UA measurements were carried out at 0, 4, 12, 24, 48 h respectively. Each measurement used a 2 mL sample. While OD_600_ was measured at 12, 24, 48 h respectively. Although NH_4_^+^ was one of the harmful by-products produced in the biomineralization process, they represented the degree of urea hydrolysis. To this end, NH_4_^+^ were measured using Nessler’s reagent colorimetric method (Whiffin et al., 2007). In addition to NH_4_^+^, the precipitation mass tended to be introduced as well for assessing the activity of the urease. Furthermore, Pb^2+^ or Cu^2+^ concentration was measured through an atomic spectrophotometer (Beijing Purkinje General Instrument TAS-990). The remediation efficiency can be evaluated *via* the equation below:

$$Remediation\;efficiency=\frac{C_{0}-C_{1}}{C_{0}}\times 100\%$$ (3)

where *C*_0_ and *C*_1_ are Pb^2+^ or Cu^2+^ concentration before and after remediation respectively. Figure 1 shows the flowchart of the test tube experiments applied to the removal of Pb or Cu using the MICP technology. Table 1 summarizes the scheme applied to the test tube experiments.

**TABLE 1:** Testing scheme applied to Pb and Cu remediation using MICP.

Contamination concentration (mM)		CaCl_2_ concentration (mM)	Urea concentration (mM)	OD_600_ values	Yeast extract (g/L)
Pb	5, 10, 30, 40, 50	250	500	1.8–2.1	2
		—	500	1.8–2.1	2
Cu	5, 10, 30, 40, 50	250	500	1.8–2.1	2
		—	500	1.8–2.1	2

### 2.3 EICP: Urease extraction

The use of *Canavalia ensiformis* mainly aimed to extract urease enzyme in the present work. The extraction method was consistent with that reported by Wang et al., 2022a; Wang et al., 2022b. *Canavalia ensiformis* was ground in the first place and sieved using a sieve with 150 μm opening. A solution composed of grounded *C. ensiformis* and ethanol was centrifuged at 8,000 r/h for a 0.5-h period and then stored at 4°C for 4 h. The supernatant extracted from the solution was centrifuged again at 4,000 r/h for a 1-h period and the precipitate was stored at −20°C. The precipitate is the urease extraction. Nessler’s reagent colorimetric method was applied to measure NH_4_^+^ concentration (Bzura and Koncki, 2019). Prior to the measurement, a calibration line was set up. The absorbance measured using a spectrophotometer was substituted into the calibration line to determine NH_4_^+^ concentration. The urease activity being measured as 342.7 U/g was categorised as low activity. In addition, the urease activity founded on the method recommended by Whiffin et al. (2007) was also calculated as 5.06 mM urea hydrolyzed min^-1^.

### 2.4 EICP: Test tube experiments

**FIGURE 2:**
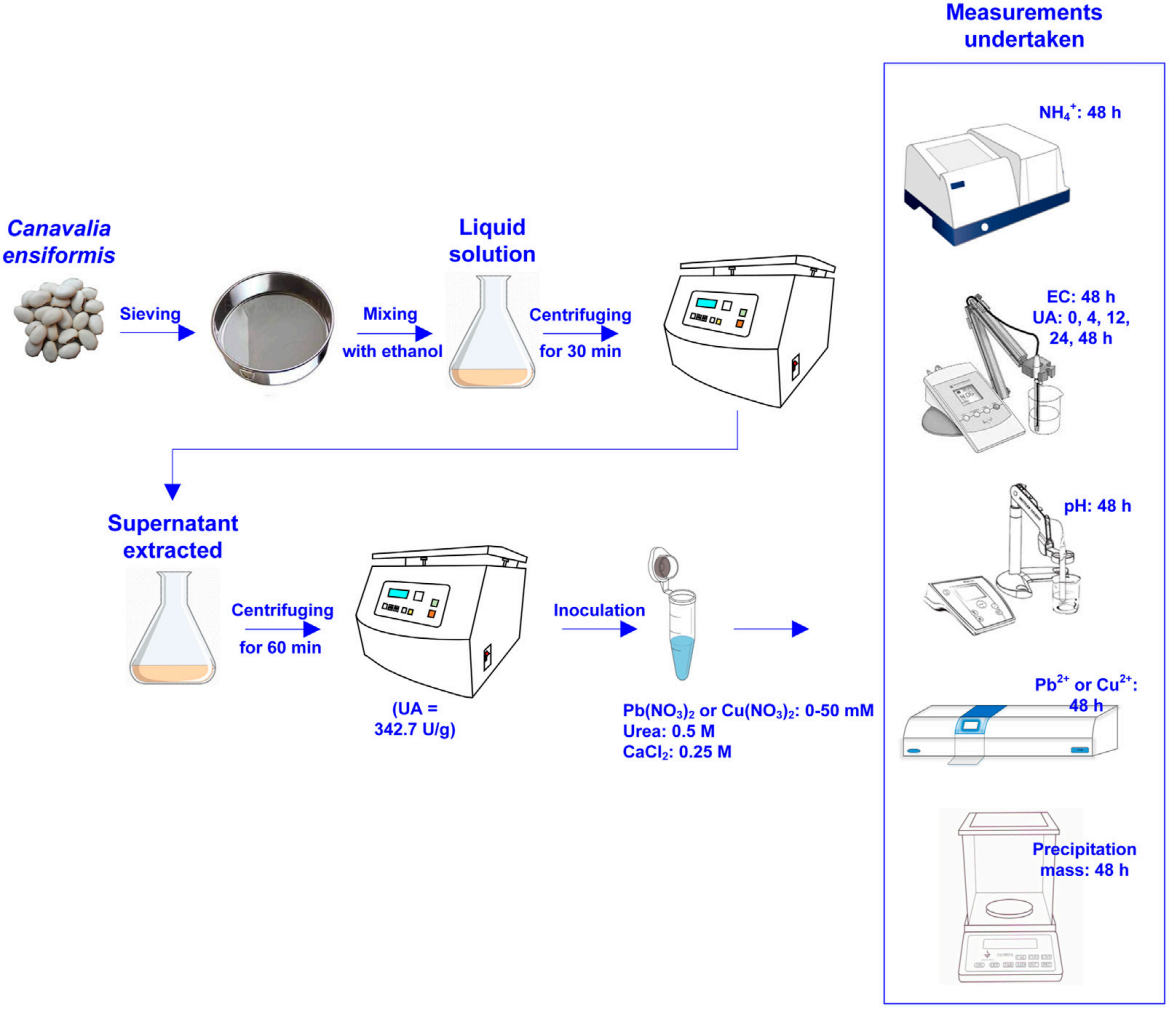
Flowchart of the test tube experiments applied to Pb or Cu remediation using the enzymatic-induced carbonate precipitation.

The test tube experiments were composed of four main phases: 1) adding urea, 2) adding Pb(NO_3_)_2_ or Cu(NO_3_)_2_, 3) adding calcium source, and 4) adding urease enzyme. Figure 2 shows the flowchart of the test tube experiments applied to the removal of Pb or Cu using the EICP technology. Measurements undertaken in the test tube experiments included pH, EC, UA, NH_4_^+^ concentration, and precipitation mass. Their frequency of measurement is summarized also in the same figure. Table 2 summarizes the scheme applied to the test tube experiments.

**TABLE 2:** Testing scheme applied to Pb and Cu remediation using EICP.

Contamination concentration (mM)		CaCl_2_ concentration (mM)	Urea concentration (mM)	Urease concentration (g/L)
Pb	5, 10, 30, 40, 50	250	500	3
		—	500	3
Cu	5, 10, 30, 40, 50	250	500	3
		—	500	3

### 2.5 Biomineralization simulation

The species and sequence of carbonate precipitation was not revealed by the test tube experiments but by the numerical simulation using the Visual MINTEQ software. The urea hydrolysis was reproduced in accordance with the ratio of NH_4_^+^ to CO_3_^2-^ being 2:1 (Gat et al., 2017) despite the omittance of bacterial cultivation and inoculation. In case CO_3_^2-^ does not play part in the biomineralization process, such a carbonate precipitation is classed as ‘abiotic’ precipitation (e.g., PbCl_2_). In contrast, it is classified as ‘biotic’ precipitation (e.g., PbCO_3_). It is well acknowledged that abiotic precipitation has a thermodynamic stability much lower than biotic precipitation, meaning that the remediation efficiency could degrade when exposed to, for example, extreme pH conditions. Considering NH_4_^+^ and CO_3_^2-^ were crucial in determining the degree of urea hydrolysis and whether the degree of urea hydrolysis is high enough to produce biotic precipitation, their concentrations were extracted upon the completion of urea hydrolysis as input parameters in the proposed numerical simulation (see Table 3). The results aimed to deepen our understanding of the sequence and species of carbonate precipitation and could be applied to explore the inherent mechanisms affecting the remediation efficiency.

**TABLE 3:** Summary of the input parameters applied to the numerical simulations.

Contamination concentration (mM)		NH_4_^+^ concentration (mM)	CO_3_^2-^ concentration (mM)	CaCl_2_ concentration (mM)	NH_4_Cl concentration (mM)
Pb	5, 10, 20, 30, 40, 50, 60, 70, 80, 90, 100	10, 30, 50, 70, 90, 110, 200	5, 15, 25, 35, 45, 55, 100	250	18.7
		10, 30, 50, 70, 90, 110, 200	5, 15, 25, 35, 45, 55, 100	—	18.7
Cu	5, 10, 20, 30, 40, 50, 60, 70, 80, 90, 100	10, 30, 50, 70, 90, 110, 200	5, 15, 25, 35, 45, 55, 100	250	18.7
		10, 30, 50, 70, 90, 110, 200	5, 15, 25, 35, 45, 55, 100	—	18.7

Note: NH_4_Cl addition was only considered in the numerical simulations under MICP.

## 3 Results and discussion

### 3.1 Effect of degree of urea hydrolysis

**FIGURE 3:**
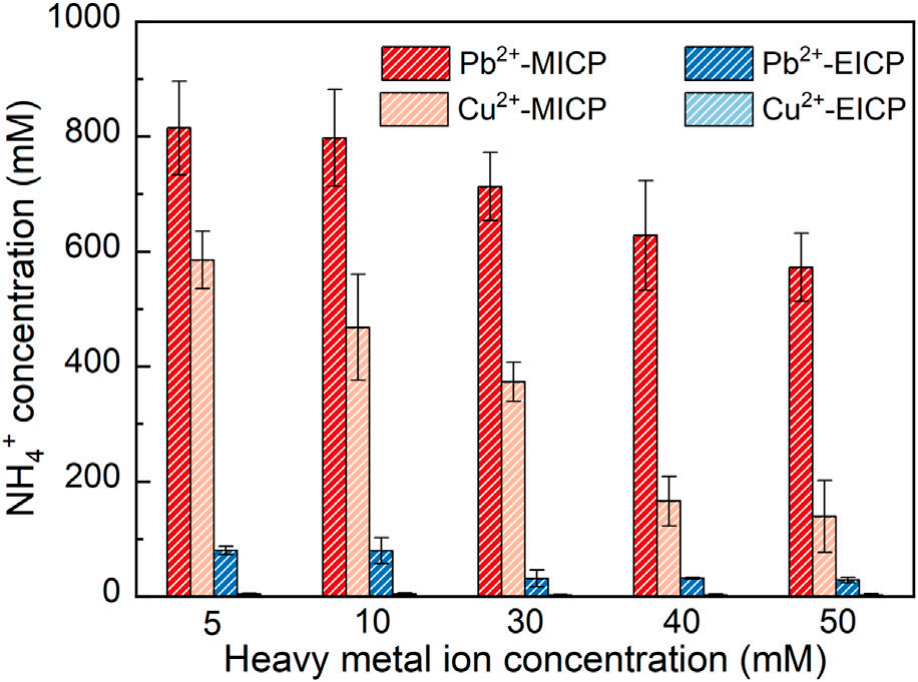
Relationships of NH_4_^+^ concentration versus Pb^2+^ or Cu^2+^ concentration under MICP and EICP respectively.

Figure 3 depicts the relationships of NH_4_^+^ concentration versus Pb^2+^ or Cu^2+^ concentration under MICP and EICP respectively. NH_4_^+^ concentration decreases with the increasing Pb^2+^ or Cu^2+^ concentration, meaning that the more significant the effect of Pb^2+^ or Cu^2+^ toxicity, the lower the urease activity, and the lower the degree of urea hydrolysis. Furthermore, for a given Pb^2+^ or Cu^2+^ concentration, NH_4_^+^ concentration under MICP is much higher than that under EICP, most likely because of UA under MICP higher than that under EICP. In this study, SUA under MICP is calculated as 8.97–10.46 mM urea hydrolyzed min^-1^ OD_600_^−1^, which is about two times higher than UA under EICP (i.e. 5.06 mM urea hydrolyzed min^-1^). These results give testimony supporting the above argument. Moreover, NH_4_^+^ concentration applied to Pb remediation is much higher than that applied to Cu remediation given their same concentration. Compared to Pb^2+^, Cu^2+^ can denature the urease, causing urease inactivation and reduction in NH_4_^+^ concentration. Given the ratio of NH_4_^+^ to CO_3_^2-^ being 2:1 (Gat et al., 2017), 100 mM NH_4_^+^ corresponding to 50 mM CO_3_^2-^ is deemed necessary to precipitate 50 mM Pb^2+^ or Cu^2+^ forming PbCO_3_ or CuCO_3_. A remediation efficiency as high as 100% could be attained in case 50 mM Pb^2+^ or Cu^2+^ is precipitated. NH_4_^+^ concentration under EICP is way below 100 mM when even subjected to 5 mM Pb^2+^ or Cu^2+^ concentration (the lowest in this work). In contrast, NH_4_^+^ concentration under MICP is in great excess of 100 mM when even subjected to 50 mM Pb^2+^ or Cu^2+^ concentration (the highest in this work). The higher NH_4_^+^ concentration under MICP is most likely due to the higher urease activity. Notwithstanding that, the remediation efficiency depends upon not only the degree of urea hydrolysis but also other influencing factors, such as species of carbonate precipitation. This would be discussed later in this paper. On the whole, NH_4_^+^ concentration decreases with the increasing Pb^2+^ or Cu^2+^ concentration. NH_4_^+^ concentration is much higher under MICP. Compared to the effect of Pb^2+^ toxicity, the effect Cu^2+^ toxicity more significantly depresses the urease activity.

### 3.2 Pb or Cu remediation

**FIGURE 4:**
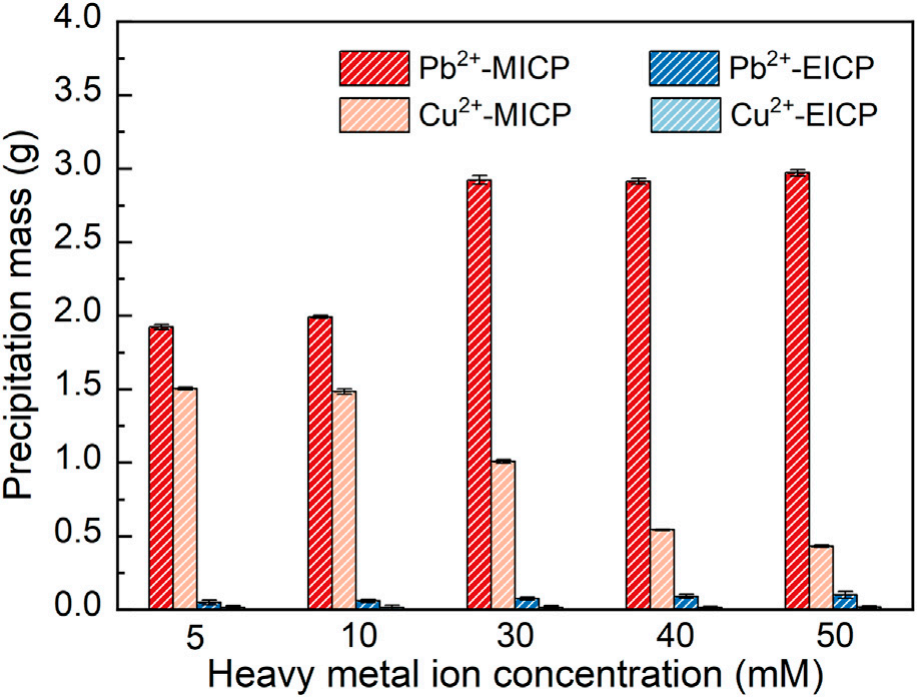
Relationships of precipitation mass versus Pb^2+^ or Cu^2+^ concentration under MICP and EICP respectively.

**FIGURE 5:**
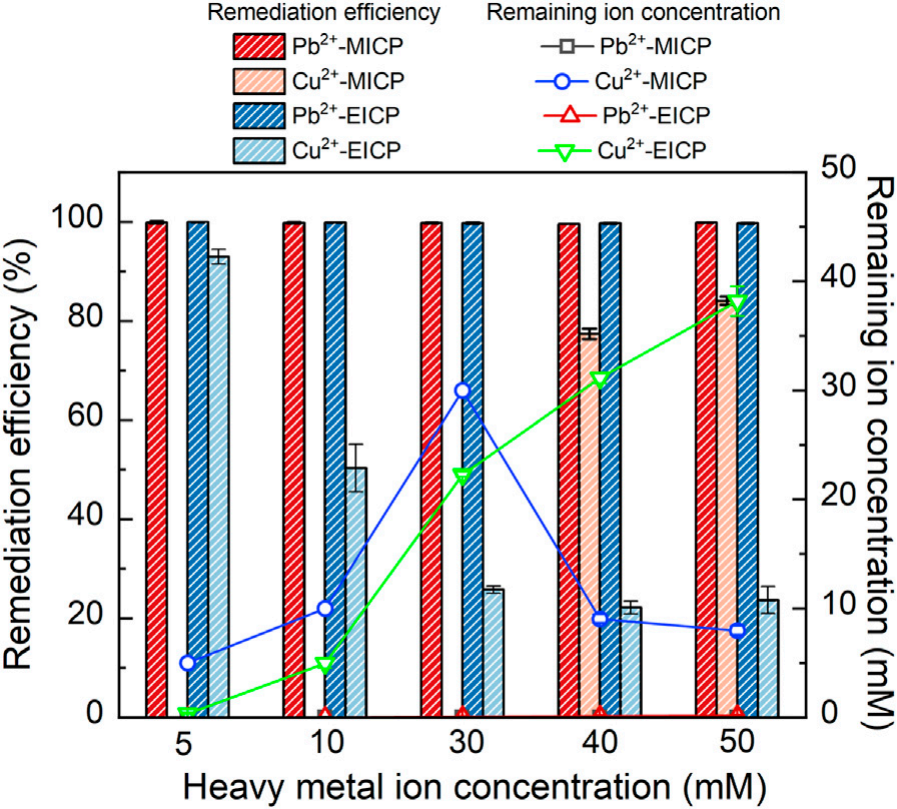
Relationships of remediation efficiency and remaining Pb^2+^ or Cu^2+^ concentration versus Pb^2+^ or Cu^2+^ concentration under MICP and EICP respectively.

The relationships of precipitation mass versus Pb^2+^ or Cu^2+^ concentration under MICP and EICP respectively are depicted in Figure 4. The precipitation mass goes up with the increase in Pb^2+^ concentration. In contrast, the precipitation mass goes down with the increasing Cu^2+^ concentration. In addition, the precipitation mass is higher under MICP than under EICP given a same Pb^2+^ or Cu^2+^ concentration. Also, the precipitation mass is higher in Pb remediation than in Cu remediation. The relationships of remediation efficiency and remaining ion concentration versus Pb^2+^ or Cu^2+^ concentration are shown in Figure 5. Under MICP, the remediation efficiency of 100% is attained by the precipitation mass of above 0.25 g when Pb^2+^ concentration falls within a 5–50 mM range. Although the precipitation mass is way below 0.25 g, the remediation efficiency of 100% is also attained under EICP when Pb^2+^ concentration falls within a 5–50 mM range. On the other hand, under MICP, the remediation efficiency of below 10% is attained by the precipitation mass of way above 1.0 g when Cu^2+^ concentration falls within a 5–30 mM range. It increases to 77% when subjected to Cu^2+^ concentration at 40 mM and further to 84% when subjected to Cu^2+^ concentration at 50 mM, in which the precipitation mass remains at about 0.5 g. Under EICP, the remediation efficiency of higher than 50% is attained by the precipitation mass of below 0.25 g when Cu^2+^ concentration falls within a range of 5–10 mM. The remediation efficiency reduces to approximately 20% when subjected to Cu^2+^ concentration falling within a range of 30–50 mM. On the whole, MICP performs similarly to EICP in terms of Pb remediation. Despite that, there is a significant discrepancy in Cu remediation between MICP and EICP. The remediation efficiency against Cu^2+^ is not as high as that against Pb^2+^, and such low remediation efficiency appears to present in some ranges of NH_4_^+^ and Cu^2+^ concentration. Also, the higher precipitation mass does not necessarily correspond to the higher remediation efficiency, although some researchers gain opposite results (Jiang et al., 2019; Wang et al., 2022). In light of this, further exploration to shed light on the impact of the species of carbonate precipitation is deemed of great necessity towards revealing the inherent mechanisms causing the reduction in the remediation efficiency against Cu^2+^.

### 3.3 Species of carbonate precipitation

**FIGURE 6:**
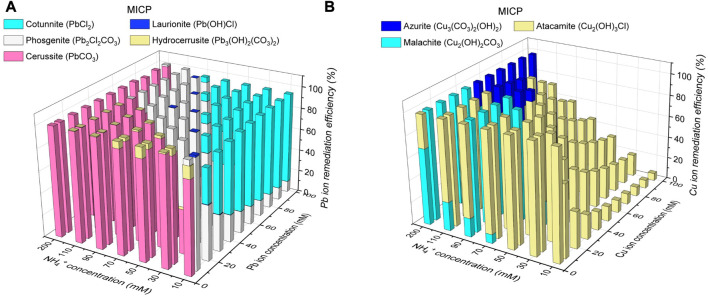
**(A)** Variations of remediation efficiency against hydrolyzed urea concentration and Pb^2+^ concentration under MICP and **(B)** variations of remediation efficiency against hydrolyzed urea concentration and Cu^2+^ concentration under MICP.

The thermodynamic stability of carbonate precipitation is deemed crucial in improving remediation efficiency. The higher the thermodynamic stability, the lower the possibility of dissolution or degradation when exposed to, for example, extreme pH conditions, and the higher the remediation efficiency. The below content presents the simulated results concerning how the remediation efficiency varies with the degree of urea hydrolysis and the species of carbonate precipitation. The variations of remediation efficiency versus hydrolyzed urea concentration and Pb^2+^ or Cu^2+^ concentration under MICP are depicted in Figure 6. When subjected to 5 mM Pb(NO_3_)_2_ and 200 mM NH_4_^+^, 100 mM CO_3_^2-^ can precipitate 100 mM Pb^2+^ (PbCO_3_) much higher than 5 mM Pb^2+^, indicating a remediation efficiency of 100% (see Figure 6A). There are four species of carbonate precipitation, including Pb_2_Cl_2_CO_3_, Pb(OH)Cl, Pb_3_(CO_3_)_2_(OH)_2_, and PbCl_2_, when CO_3_^2-^ concentration is not high enough to precipitate Pb^2+^. The Raman peak at 150 cm^-1^ and 1,056 cm^-1^ corresponds to the stretching vibration of CO_3_^2-^ when it binds to Pb^2+^ (see ‘red’ line in Figure 8A. Three tensile vibrations at 60 cm^-1^, 150 cm^-1^, and 1,056 cm^-1^ are the footprint of their show up (i.e., PbCl_2_ and Pb_2_Cl_2_CO_3_). Also, one tensile vibration is recorded by Raman spectra at 106 cm^-1^, corresponding to the presence of Pb(OH)Cl and Pb_3_(CO_3_)(OH)_2_. 5 mM CO_3_^2-^ can only precipitate 10 mM Pb^2+^ (Pb_2_Cl_2_CO_3_) when subjected to 50 mM Pb(NO_3_)_2_ and 10 mM NH_4_^+^, leaving 40 mM Pb^2+^ behind. The remaining 40 mM Pb^2+^ can only be precipitated with Cl^−^ toward reducing the remediation efficiency by 9%. Also, the reduction in CO_3_^2-^ concentration causes a difficulty in securing the remediation efficiency because Cu remediation is not attained through biotic precipitation but through abiotic precipitation. For example, when NH_4_^+^ concentration is reduced sharply from 200 mM to 10 mM and Cu(NO_3_)_2_ concentration is notably elevated from 10 mM to 100 mM, Cu_2_CO_3_(OH)_2_ is transformed to Cu_2_(OH)_3_Cl, causing a reduction of the remediation efficiency by 93.3% (see Figure 6B). Despite that, the other two species of precipitation (Cu_3_(CO_3_)_2_(OH)_2_ and Cu_2_(OH)_2_CO_3_) are present when CO_3_^2-^ concentration is high enough. The adsorption band at 1,437–1,548 cm^-1^ range is mainly attributed to the formation of Cu_3_(CO_3_)_2_(OH)_2_ (see ‘red’ line in Figure 8B). The Raman peak at 1,008 cm^-1^, 1,437 cm^-1^, and 3,356 cm^-1^ is responsible for the precipitation of Cu_3_(CO_3_)_2_(OH)_2_. Also, the adsorption band at 77–509 cm^-1^ range and 3,356–3,441 cm^-1^ range presents strong correspondence with the presence of Cu_2_(OH)_3_Cl. Cu_2_(OH)_2_CO_3_ is precipitated when subjected to 10 mM Cu(NO_3_)_2_ and 200 mM NH_4_^+^.100 mM CO_3_^2-^ can precipitate 200 mM Cu^2+^ much higher than 10 mM Cu^2+^, indicating a remediation efficiency of 100%.

**FIGURE 7:**
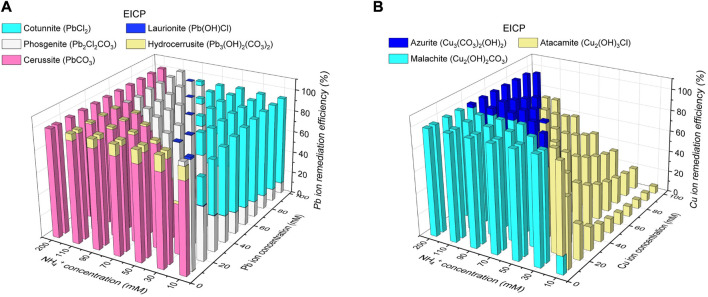
**(A)** Variations of remediation efficiency against hydrolyzed urea concentration and Pb^2+^ concentration under EICP and **(B)** variations of remediation efficiency against hydrolyzed urea concentration and Cu^2+^ concentration under EICP.

**FIGURE 8:**
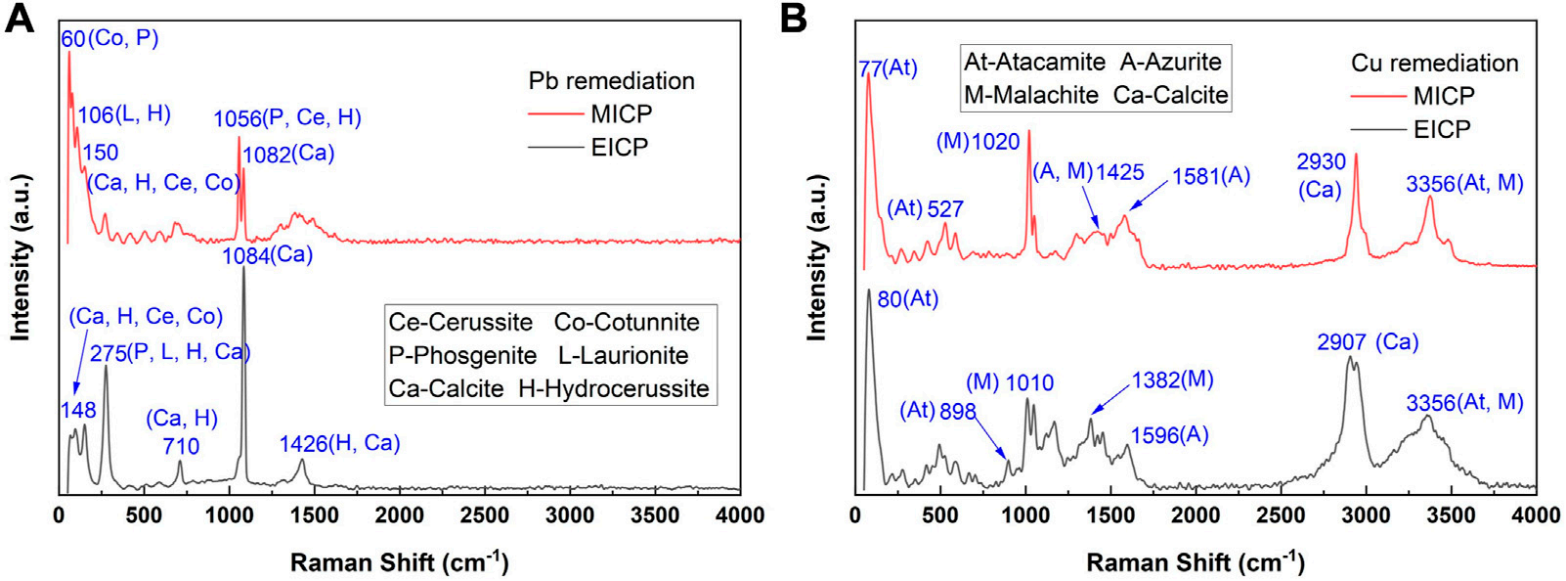
Raman spectra of samples taken under EICP and MICP: **(A)** Pb remediation and **(B)** Cu remediation.

The variations of remediation efficiency versus hydrolyzed urea concentration and Pb^2+^ or Cu^2+^ concentration under EICP are illustrated in Figure 7 When subjected to 5 mM Pb(NO_3_)_2_ and 200 mM NH_4_^+^, 100 mM CO_3_^2-^ can precipitate 100 mM Pb^2+^ (PbCO_3_) much higher than 5 mM Pb^2+^, corresponding to a remediation efficiency of 100% (see Figure 7A). PbCl_2_, Pb(OH)Cl, Pb_2_Cl_2_CO_3_, and Pb_3_(OH)_2_(CO_3_)_2_ are precipitated when CO_3_^2-^ concentration is not high enough. The Raman peaks under EICP are comparable with those under MICP, and to prevent repetition, their interpretation is neglected here (see ‘black’ line in Figure 8A). PbCO_3_, when subjected to 100 mM Pb(NO_3_)_2_ and 10 mM NH_4_^+^, is transformed to PbCl_2_ and Pb_2_Cl_2_CO_3_ because 5 mM CO_3_^2-^ can only precipitate 10 mM Pb^2+^ (Pb_2_Cl_2_CO_3_), leaving 90 mM Pb^2+^ to be precipitated with Cl^−^. The formation of PbCl_2_ reduces the remediation efficiency by about 8%. As to Cu remediation, Cu_2_(OH)_2_CO_3_ could be transformed to Cu_3_(CO_3_)_2_(OH)_2_ and Cu_2_(OH)_3_Cl when the degree of urea hydrolysis is not as high as expected. The Raman peaks under EICP are generally in line with those under MICP (see ‘black’ line in Figure 8B). Given 100 mM Cu(NO_3_)_2_ and 10 mM NH_4_^+^, the formation of Cu_2_(OH)_3_Cl leads to a substantial reduction in the remediation efficiency by 93.3% (see Figure 7B). In contrast, 100 mM CO_3_^2-^ can precipitate 200 mM Cu^2+^ (Cu_2_(OH)_2_CO_3_) when subjected to 5 mM Cu(NO_3_)_2_ and 200 mM NH_4_^+^, meaning that a majority of Cu^2+^ is precipitated with a remediation efficiency of 100%. The initially formed abiotic precipitates improve the resistance to Pb or Cu toxicity and helps the conversion into biotic precipitates which form the outer layer and encapsulate the initial abiotic precipitates (Jiang et al., 2019). Similar precipitate conversions can also be seen in the work done by Achal et al. (2012). These results lead us to summarize that the remediation efficiency against Pb^2+^ or Cu^2+^ could not only be influenced by the degree of urea hydrolysis but by the species of carbonate precipitation. The low degree of urea hydrolysis leads to low CO_3_^2-^ concentration, promoting the formation of abiotic precipitation, such as PbCl_2_ and Cu_2_(OH)_3_Cl. They degrade notably the remediation efficiency.

### 3.4 Chemical and thermodynamic properties of carbonates

**FIGURE 9:**
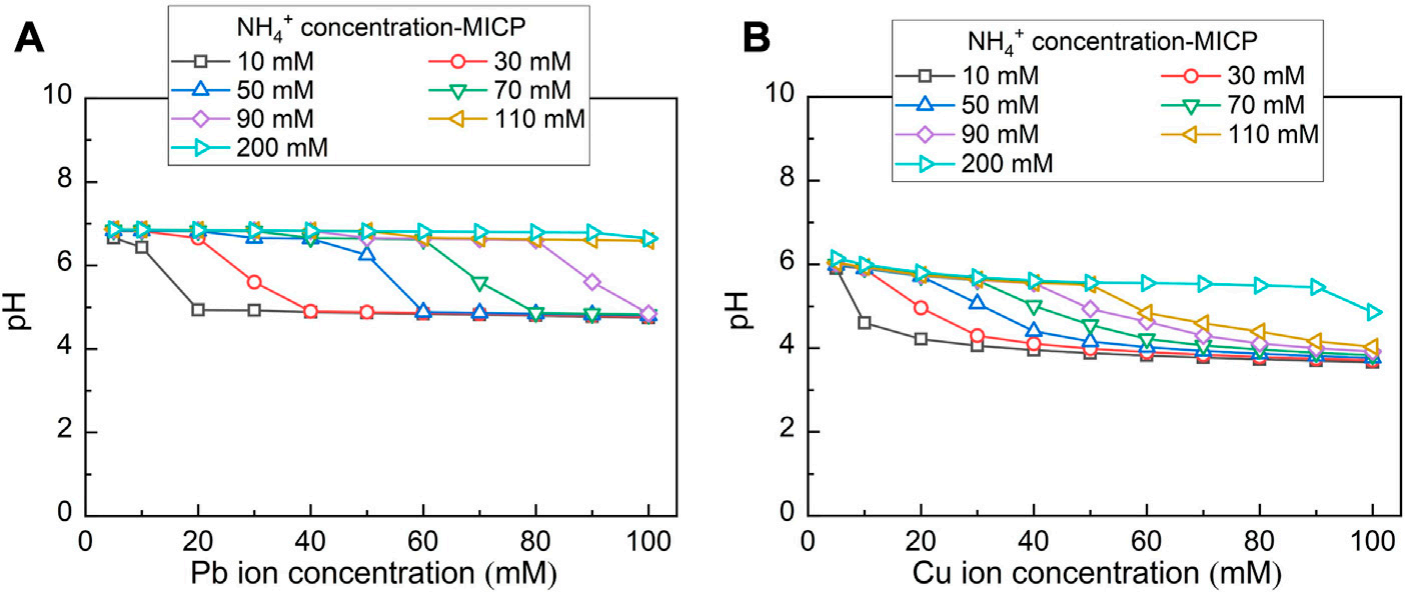
**(A)** Variations of pH surrounding against Pb^2+^ concentration under MICP and **(B)** variations of pH surrounding against Cu^2+^ concentration under MICP.

**FIGURE 10:**
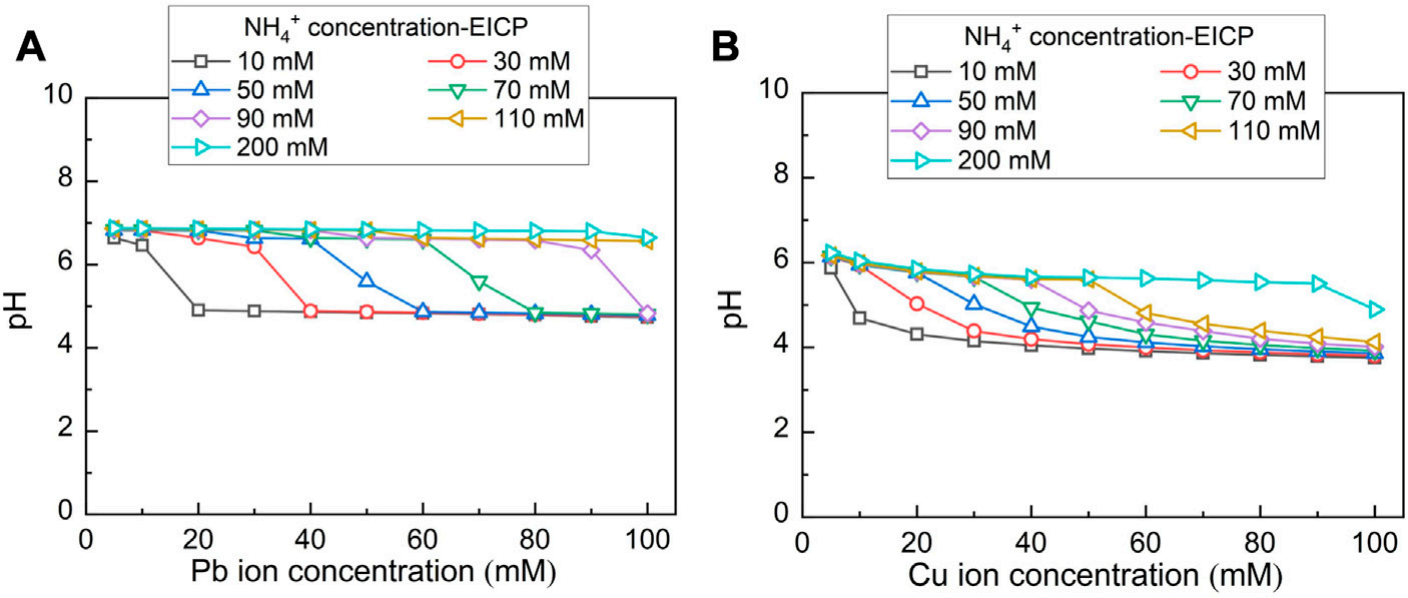
**(A)** Variations of pH surrounding against Pb^2+^ concentration under EICP and **(B)** variations of pH surrounding against Cu^2+^ concentration under EICP.

Chemical stability for carbonate precipitations under harsh pH conditions is deemed crucial in securing remediation efficiency. The variation of pH against Pb^2+^ and Cu^2+^ under MICP and EICP considering the hydrolysed urea concentrations varying in a 10–200 mM range is depicted in Figures 9, 10 respectively. pH, while remedying Pb^2+^ using MICP, reduces from 7 to 5 (see Figure 9A). Higher NH_4_^+^ concentrations delay such reduction in pH when Pb^2+^ concentration goes up. pH remains at some 7 when NH_4_^+^ concentration reaches 200 mM. pH, while remedying Cu^2+^ using MICP, reduces from 6 to 4 (see Figure 9B). Similarly, higher NH_4_^+^ concentrations put off such reduction in pH as Cu^2+^ concentration goes up. While remedying Cu^2+^, pH being approximately 6 is attained as NH_4_^+^ concentration reaches 200 mM. When subjected to such harsh pH conditions, carbonate precipitation of low chemical stability, induced by MICP, may dissolve or degrade. Lower degrees of urea hydrolysis could aggravate the dissolution or degradation of carbonate precipitation. On the other hand, while remedying Pb^2+^ using EICP, pH decreases from 7 to 5 (see Figure 10A). Further, pH decreases from 6 to 4 while remedying Cu^2+^ using EICP (see Figure 10B). It can also be seen that higher NH_4_^+^ concentrations retard such reduction in pH when Pb^2+^ or Cu^2+^ concentration is lifted up. The simulated results show that under either MICP or EICP, the lower degrees of urea hydrolysis correspond to cotunnite and atacamite precipitations and also to a reduction in the remediation efficiency (see Figures 6, 7). Under some circumstances, the remediation efficiency corresponding to atacamite precipitation could be as low as below 10%. In light of this, cotunnite and atacamite’s chemical stability are considered lower compared to calcite, cerussite, phosgenite, and malachite.

Given a biochemical system undergoing a reversible reaction, a thermodynamic equilibrium constant, K, is defined to be the value of the reaction quotient when forwards and reverse reactions take place at the same time. The higher the K value, the higher the transformation potential of carbonate precipitation to another phase, and the lower the thermodynamic stability. If the composition of a chemical precipitation at equilibrium is changed by addition of some chemical reagent, a new equilibrium will be attained, given enough time. K is related to the composition of the carbonate precipitation at equilibrium by Eqs. 4 and 5.

$$K=\frac{{\left\{R\right\}}^{\rho }{\left\{S\right\}}^{\sigma }\ldots }{{\left\{A\right\}}^{\alpha }{\left\{B\right\}}^{\beta }\ldots }=\frac{{\left[R\right]}^{\rho }{\left[S\right]}^{\sigma }\ldots }{{\left[A\right]}^{\alpha }{\left[B\right]}^{\beta }\ldots }\times \Gamma$$ (4)

$$\Gamma =\frac{\gamma_{R}^{\rho }\gamma_{S}^{\sigma }\ldots }{\gamma_{A}^{\alpha }\gamma_{B}^{\beta }\ldots }$$ (5)

where {X} represents the thermodynamic activity of reagent X at equilibrium, [X] the numerical value of the corresponding concentration in moles per litre, Γ the quotient of activity coefficient, and γ the corresponding activity coefficient. Assuming the value of Γ is constant over a range of experimental conditions, such as pH, an equilibrium constant can be derived as a quotient of concentrations (see Eq. 6).

$$K_{c}=\frac{K}{\Gamma }=\frac{{\left[R\right]}^{\rho }{\left[S\right]}^{\sigma }\ldots }{{\left[A\right]}^{\alpha }{\left[B\right]}^{\beta }\ldots }$$ (6)

As K is linked to the standard Gibbs free energy change of reaction ∆G, their relationship can be expressed by Eq. 7.

**FIGURE 11:**
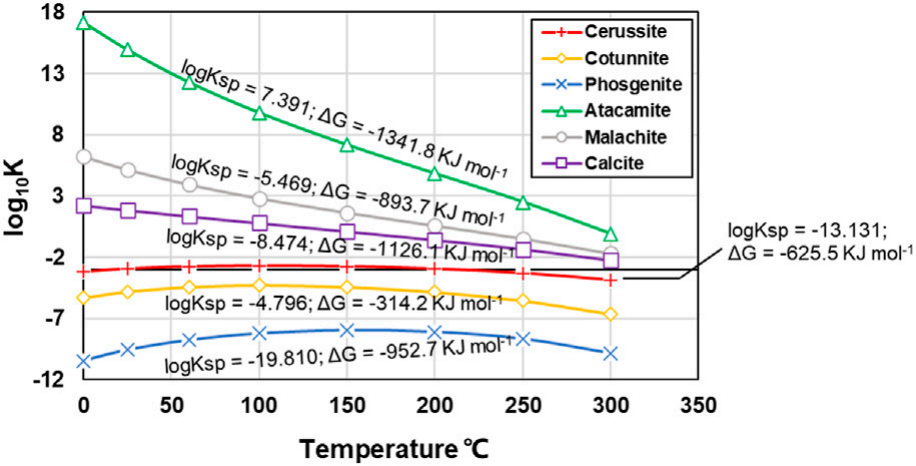
Relationships of log_10_K (thermodynamic equilibrium constant) versus temperature (in Celsius) against different carbonate precipitations applied to Pb and Cu remediation.

∆G=−RTln K (7)

where R represents the universal gas constant, T the absolute temperature (in Kelvins), and ln the natural logarithm. Given ∆G and R known (Benson and Teague, 1980; Robie and Hemingway, 1995; Blanc, 2017), the relationships of log_10_K versus T (in Celsius) for the six carbonate precipitations in the simulated results are depicted in Figure 11. Amongst the six carbonate precipitations, cotunnite (PbCl_2_) and atacamite (Cu_2_(OH)_3_Cl) are categorized as the abiotic precipitation, while calcite (CaCO_3_), cerussite (PbCO_3_), phosgenite (Pb_2_Cl_2_CO_3_), and malachite (Cu_2_(OH)_2_CO_3_) are classed as the biotic precipitation. Log_10_K for atacamite decreases from 17.16 to −0.10 when the temperature is increased from 0 to 300 deg. Further, for a given T, log_10_K for atacamite is the highest amongst the six carbonate precipitations. Moreover, the solubility product Ksp being about 7.391 for atacamite is also the highest amongst the six carbonate precipitations. The higher the Ksp, the more difficult the formation of carbonate precipitation. These results indicate that atacamite has the highest potential of transforming to another phase when subjected to a change in temperature, thus indicating a reduction in the thermodynamic stability (the lowest in the present work). On the other hand, cotunnite has the second highest Ksp, although for a given temperature, its log_10_K is the second last. Also, log_10_K for cotunnite does not show substantial change as the temperature is increased from 0 to 300 deg. Notwithstanding that, cotunnite is still considered to be unstable when such high Ksp promotes the potential of transforming to another phase during biochemical processes. The thermodynamic properties of calcite are also dominated by the competition between calcite and aragonite structures in the crystalline state (Radha and Navrotsky, 2013; Xu et al., 2020). Such a mineralogical analysis will be discussed in another paper. It is worth noting that for the test tube experiments, more than 400 mM NH_4_^+^ are hydrolysed when subjected to Cu^2+^ concentration falling in a 5–30 mM range, but such high NH_4_^+^ concentration corresponds to a remediation efficiency close to zero (see Figure 3; Figure 5). Although NH_4_^+^ concentration higher than 400 mM is not within the scope of the numerical simulation, the remediation efficiency close to zero is most likely due to the fact that NH_4_^+^ concentration higher than 400 mM raises pH to above 9, and such strongly alkaline environments promote the formation of copper-ammonia complex with a chemical formula of [Cu(NH_3_)_4_(H_2_O)_2_]^2+^ (Duarte-Nass et al., 2020; Wang et al., 2023a). The copper-ammonia complex turns Cu^2+^ into a free state degrading the remediation efficiency. These results lead us to conclude that a reduction in the remediation efficiency could be either due to lower degrees of urea hydrolysis or to carbonate precipitation with low chemical and thermodynamic properties. Apart from that, a reduction, while remedying Cu^2+^, could also be present due to the formation of the copper-ammonia complex.

## 4 Conclusions

This paper presented the results concerning the immobilization of Pb and Cu in aqueous solution using MICP and EICP respectively, highlighting their relative merits. Based on the results and discussion, some main conclusions can be drawn as follows.

(1) During the biomineralization process, NH_4_^+^ concentration decreases with the increasing Pb^2+^ or Cu^2+^ concentration, and for a given Pb^2+^ or Cu^2+^ concentration, it is much higher under MICP. The remediation efficiency against Pb^2+^ using MICP performs similarly to that using EICP. However, a discrepancy in the remediation efficiency against Cu^2+^ between MICP and EICP is observed. Further, the remediation efficiency against Cu^2+^ is way below that against Pb^2+^.
(2) The immobilization of Pb or Cu is attained through biotic precipitation when CO_3_^2-^ concentration is high enough to precipitate the majority of Pb^2+^ or Cu^2+^. In case CO_3_^2-^ concentration is not high enough, it is attained *via* abiotic and biotic precipitations, corresponding to a reduction in the remediation efficiency. Further, high precipitation mass does not necessarily correspond to high remediation efficiency.
(3) The reduction in the remediation efficiency appears to relate to the chemical and thermodynamic properties of the carbonate precipitations. Results indicate that the reduction in the remediation efficiency is ascribed to two precipitates (i.e., cotunnite and atacamite). Their degradation may take place when subjected to harsh pH conditions or a substantial change in temperature, thus reducing the remediation efficiency. In addition, the reduction in the remediation efficiency may also attribute to the formation of copper-ammonia complex when higher NH_4_^+^ concentrations raise pH to above 9.

## Data Availability

The original contributions presented in the study are included in the article/supplementary material, further inquiries can be directed to the corresponding author.
